# Application of plasma donor-derived cell free DNA for lung allograft rejection diagnosis in lung transplant recipients

**DOI:** 10.1186/s12890-022-02229-y

**Published:** 2023-01-26

**Authors:** Chunrong Ju, Xin Xu, Jianheng Zhang, Ao Chen, Qiaoyan Lian, Feng Liu, Haitao Liu, Yuhang Cai, Yanjun Zou, Yalan Yang, Yang Zhou, Jianxing He

**Affiliations:** 1 State Key Laboratory of Respiratory Disease, National Clinical Research Center for Respiratory Disease, Guangzhou Institute of Respiratory Health, The First Affiliated Hospital of Guangzhou Medical University, Guangzhou, 510000 China; 2 AlloDx (Shanghai) Biotech., Co., Ltd, Shanghai, 201100 China; 3 School of Life Sciences, Jiangsu University, Zhenjiang, 212013 China

**Keywords:** Lung transplantation, Cell-free DNA, Acute rejection, Infection, Chronic lung allograft dysfunction

## Abstract

**Background:**

Donor-derived cell-free DNA (dd-cfDNA) has been applied to monitor acute rejection (AR) in kidney and heart transplantation. This study was aimed to investigate the application of dd-cfDNA levels in the diagnosis of AR and chronic lung allograft dysfunction (CLAD) among the lung transplantation recipients (LTRs).

**Methods:**

One hundred and seventy LTRs were enrolled at the First Affiliated Hospital of Guangzhou Medical University between 1 June 2015 and 30 March 2021. Patients were divided into 4 groups: stable group, AR group, infection group and CLAD group. The level of dd-cfDNA was analyzed using target region sequencing and the performance characteristics of dd-cfDNA for diagnosis of AR and CLAD were determined, respectively.

**Results:**

Kruskal–Wallis test showed that there were some significant differences in the level of dd-cfDNA (%) among the 4 groups, with *p* < 0.001. Among them, the level of dd-cfDNA (%) was highest (median 2.17, IQR [1.40–3.82]) in AR group, and higher in CLAD group (median 1.07, IQR [0.98–1.31]), but lower in infection group (median 0.71, IQR [0.57–1.07]) and lowest in stable group (median 0.71, IQR [0.61–0.84]). AUC-ROC curve analysis showed that the threshold of dd-cfDNA for AR was 1.17%, with sensitivity being 89.19% and specificity being 86.47%, and the optimal threshold of 0.89% was determined of CLAD, with sensitivity being 95.00% and specificity of 76.99%.

**Conclusions:**

Plasma dd-cfDNA could be a useful tool for the assessment of lung allograft rejection, including AR and CLAD, and holds promise as a noninvasive biomarker for “allograft injury” in both acute and chronic rejection following lung transplantation.

**Supplementary Information:**

The online version contains supplementary material available at 10.1186/s12890-022-02229-y.

## Background

Lung transplantation, at present, is the only therapeutic option for patients with end-stage lung or pulmonary vascular disease. Lung transplantation has witnessed dramatic growth worldwide in the recent years; over 4,000 lung transplants are performed annually worldwide [1]. Nonetheless, the survival rate following lung transplantation is the lowest among all solid-organ transplants (11 y for heart and 8.5 y for liver) [2–4]. Lung transplant patients have the shortest survival of any other solid organ transplantation, primarily because of the high incidence of chronic lung allograft dysfunction (CLAD) [5, 6]. Other pulmonary complications, such as persistent rejection and severe infection, also increase mortality among lung transplant recipients [7]. However, CLAD remains the main limiting factor for reduced life expectancy after lung transplantation, occurring in approximately 50% of transplant recipients within 5 years of primary lung transplantation [6, 8]. Approximately 27% of recipients experience at least one episode of treated acute cellular rejection (ACR) during the initial year post-lung transplantation. Antibody-mediated rejection (AMR) is also a very severe complication which leads to allograft lung injury and increase patient mortality. AR increases the mortality significantly; moreover, AR is well recognized as a risk factor for the development of bronchiolitis obliterans syndrome (BOS), the major form of CLAD. Increased severity of AR and number of episodes of AR increase the risk of BOS [9], but also leads to a prior CLAD. CLAD is the leading cause of death for lung transplant recipients (LTRs) after 1-year post-lung transplantation. CLAD represents a progressive and irreversible type of tissue injury that ultimately culminates in allograft failure. Therefore, exact detection of AR and CLAD in the early stage is critical for mitigating lung transplantation mortality.

Currently, pathology from invasive biopsy tissue is the golden standard for diagnosis of AR and CLAD. Among the procedures commonly used to get the lung tissues, trans-bronchial lung biopsies (TBLBs) are the first choice. However, this approach is difficult to practice for most cases in clinical settings. Apart from the potential risks, cost-prohibitive burden, and inconvenience, it is always difficult to get a consent from the patients mainly because it is an invasive procedure; also, physicians may care about minimizing the development of secondary complications in relation to TBLB and may thus be unwilling to perform the procedure. Moreover, biopsies are costly, sampling-error prone, and also have restricted specificity and sensitivity, limited by subjective interpretation [10]. Thus, a non-invasive, relatively objective, and specific biomarker is urgently required for examining AR and CLAD complications.

Recently, plasma donor derived cell-free DNA (dd-cfDNA) has become a hot topic in field of solid organ transplantation because emerging evidence suggests that detection of allograft injury via dd-cfDNA may be a noninvasive biomarker for allograft rejection [11–17]. Moreover, dd-cfDNA quantification has been explored as a novel means of monitoring pulmonary rejection in lung transplant recipients plasma internationally [18]. However, dd-cfDNA detection has not yet been applied in Chinese LTRs. In the current work, we investigate the application level of dd-cfDNA in the diagnosis of ACR and CLAD among the LTRs.

## Materials and methods

### Study design and population

A non-experimental retrospective cross-sectional study design was used in this work. This study adhered to the tenets of the Declaration of Helsinki and was approved by the ethics committee of the First Affiliate Hospital of Guangzhou Medical University (k2021-98). The informed consent was obtained from each recipient.

The recipients who had undergone lung transplantation at the First Affiliated Hospital of Guangzhou Medical University between 1 June 2015 and 30 March 2021 were screened. The recipients who underwent blood sample collection and dd-cfDNA testing were enrolled in our center from May 2020 to December 2021. The sequence data reported in this paper have been deposited in the Sequence Read Archive, https://dataview.ncbi.nlm.nih.gov/object/PRJNA801094?reviewer=vap7nq272jc2l86ligrr7clm3m (accession no. PRJNA801094). The inclusion criteria were as follows: 1) age ≥ 18 years; 2) single- or double-lung transplantation; 3) more than 14 days from the day of transplantation. The exclusion criteria were as follows: 1) incomplete medical data; 2) combined heart–lung transplantation or multi-organ transplantation; 3) “mixed diagnosis” of concurrent infection with rejection; 4) died from unknown causes.

Study cohorts were divided into 4 groups based on the final diagnosis which was determined by combining the clinical information and review of the treatment response. The study participants were divided into the following 4 groups: stable group (stable state without infection or rejection), AR group (biopsy-confirmed or clinically treated AR without biopsy confirmation; including AMR and grades A1-A4 ACR), infection group (lung allograft infection without concurrent rejection), and CLAD group (CLAD with or without risk factors).In the stable group, no symptoms were observed at the time of sample collection. In the infection group, the patients had some symptoms, which consisted of a new-onset cough or expectoration or aggravation of existing respiratory tract symptoms with or without purulent sputum, chest pain, dyspnea, hemoptysis, headache, fever, and rales on lung auscultation. In the AR group, the patients also had the similar symptoms mentioned above as in the infection group, and adventitious sounds on lung auscultation. In the CLAD group, most patients had decreased exercise capacity, shortness of breath, dyspnea (on exertion or at rest) to a varying degree, dry cough, or slight chest tightness that had persisted or progressed for at least 3 weeks.

### Clinical assessment

Data of study participants that were collected retrospectively, including demographic data, primary indication for lung transplantation, type of surgery (unilateral or bilateral lung transplantation), operation-related data, plasma cytomegalovirus (CMV) IgG status, CMV-DNA loads were record for both donor and recipients, induction treatment regimen, panel reactive antibody (PRA) and donor specific antibody (DSA) prior to and after the transplantation for the recipients, clinical information such as symptoms, signs, chest imaging findings such as X-ray or computed tomography (CT), laboratory routine tests, biochemical tests, tacrolimus trough levels, and etc.

There were no opacities on the CT scans in the stable group. In the CLAD group, patients with BOS had air trapping/mosaic attenuation on their expiratory CT scan, whereas a common denominator in patients with restrictive allograft syndrome (RAS) was the presence of persistent pleuroparenchymal infiltrates on CT imaging.

### Immunosuppressive induction treatment at the time of LTx

As part of our routine clinical protocol, all lung transplant recipients were treated with standardized triple immunosuppressive regimens comprising of calcineurin inhibitors (tacrolimus or cyclosporin A), mycophenolate mofetil, and prednisolone. Most patients were treated with basiliximab to induce immunosuppression.

### Detection of dd-cfDNA in blood samples

For dd-cfDNA detection, we specifically selected plasma samples more than 14-days posttransplant, to avoid the potential confounding complication of lung injury from ischemic-reperfusion of lung transplant operation. Plasma samples were associated with concurrent histopathologic diagnoses and bronchial-lavage fluid (BALF) microbiologic cultures. Samples that passed strict quality control from unique patients and had adequate plasma sample volume (2 mL) were analyzed with a clinical-grade NGS dd-cfDNA assay at a Chinese-certified Laboratory (AlloDx Biotech, Co., Ltd).

A total of 6,200 human SNP loci were enriched by liquid hybridization, SNP loci selection criteria and bioinformatics protocol was conducted as reported in our previous study [19–21]. In short, blood (8 mL) was drawn into cfDNA blood collection tubes (Streck, Omaha, NE, USA). Plasma was separated by centrifugation at 1,600 × g for 10 min followed by a second centrifugation at 16,000 × g for 10 min, and cfDNA was immediately extracted from 1.8 mL plasma using the Circulating Nucleic Acid kit (Qiagen, Cat.No55114, Germany). 30 ng cfDNA was used in this study to construct a sequencing library for next generation sequencing (KAPA LTP Library Preparation Kit, KK8235, Roch, Switzerland), and captured libraries were then sequenced on an Illumina (X-ten, 10 ± 5 million, PE 150 bp). Sequencing of raw data was processed using BWA and Samtools. Bayes approach was applied to quantify the dd-cfDNA level.

### Histopathology from lung biopsy tissue

The lung lobes with the most prominent lesions according to chest CT were selected for TBLB with fiberoptic bronchoscopy by using standardized procedures. Histopathology for TBLB specimens were evaluated by two experienced pathologists according to the Revised International Society of Heart and Lung Transplant (ISHLT) working group recommendations [22]. In addition, the peripheral blood samples were collected for dd-cfDNA assay on the day of the biopsy or within two days before the biopsy. No sample collection was performed for patients within three days after the methylprednisolone pulse therapy, or within two days after the lung biopsy, and for patients undergoing vigorous exercise in recent hours. ACR was graded for lung tissues from TBLB according to the Revised ISHLT Histopathological Classification as: Grade A (perivascular lymphocytic infiltration) subtypes A0: absence of ACR; A1: minimal; A2: mild; A3: moderate; A4: severe; Grade B (lymphocytic bronchiolitis) subtypes B0-2R, BX; and Grade C (presence or absence of bronchiolitis obliterans)_._ AMR was determined by the ISHLT and Banff Lung pathology working group criteria, which were consistent with “probable” AMR [23]. BOS was determined by ISHLT consensus council guidelines for classification of CLAD [24, 25]. The diagnosis of AR (without histological proof) was ascertained by a panel of 3 experienced transplant clinicians according to the comprehensive clinical assessments and reviewing the treatment response. The comprehensive assessments consisted of the clinical presentations, a lung function decrease by more than 10% in forced vital capacity (FVC) and forced expiratory volume in 1 s (FEV1), positive HRCT manifestations (ground glass opacity, interlobular septal thickening, with or without pleural effusion, etc.) [26], biochemical test results, cellular and immunological indices, and exclusion of other causes such as infection. Additionally, DSA should be present for AMR. Pulmonary infection was diagnosed based on our previous studies [27, 28]. There were no pathogens in the AR or CLAD groups. There were different pathogens present in the infection group. We did not collect BALF samples from the recipients in the stable group as they were in a stable state with no symptoms or opacities on the CT scan.

### Statistical analyses

All analyses were performed in IBM SPSS Statistics (V.22). Graphs were created using GraphPad Prism 8 for Windows. Descriptive statistics are used to describe the LTRs demographics and distribution of dd-cfDNA measurements obtained from blood samples. Non-parametric distributions were compared using the Wilcoxon rank sum test (two groups), and the Kruskal–Wallis rank sum test (multiple groups). Post-hoc analysis for the latter was performed using Dunn’s test with Bonferroni adjustment. Paired t test was used to compare the change rate of FEV1%pred. Spearman's rank correlation was used to assess relationships between dd-cfDNA (%) and the rate of lung function change. A receiver operating characteristic curve (ROC) analysis was performed to assess how well dd-cfDNA fraction (%) discriminated among different subgroup. A threshold was established to categorize dd-cfDNA scores indicating AR, CLAD or demonstrating stable or infection based upon the distribution of the data. All statistical analyses were two-tailed, and *p*-level less than 0.05 is considered a significant difference.

## Results

### Demographic characteristic of the study participants

**Fig. 1:**
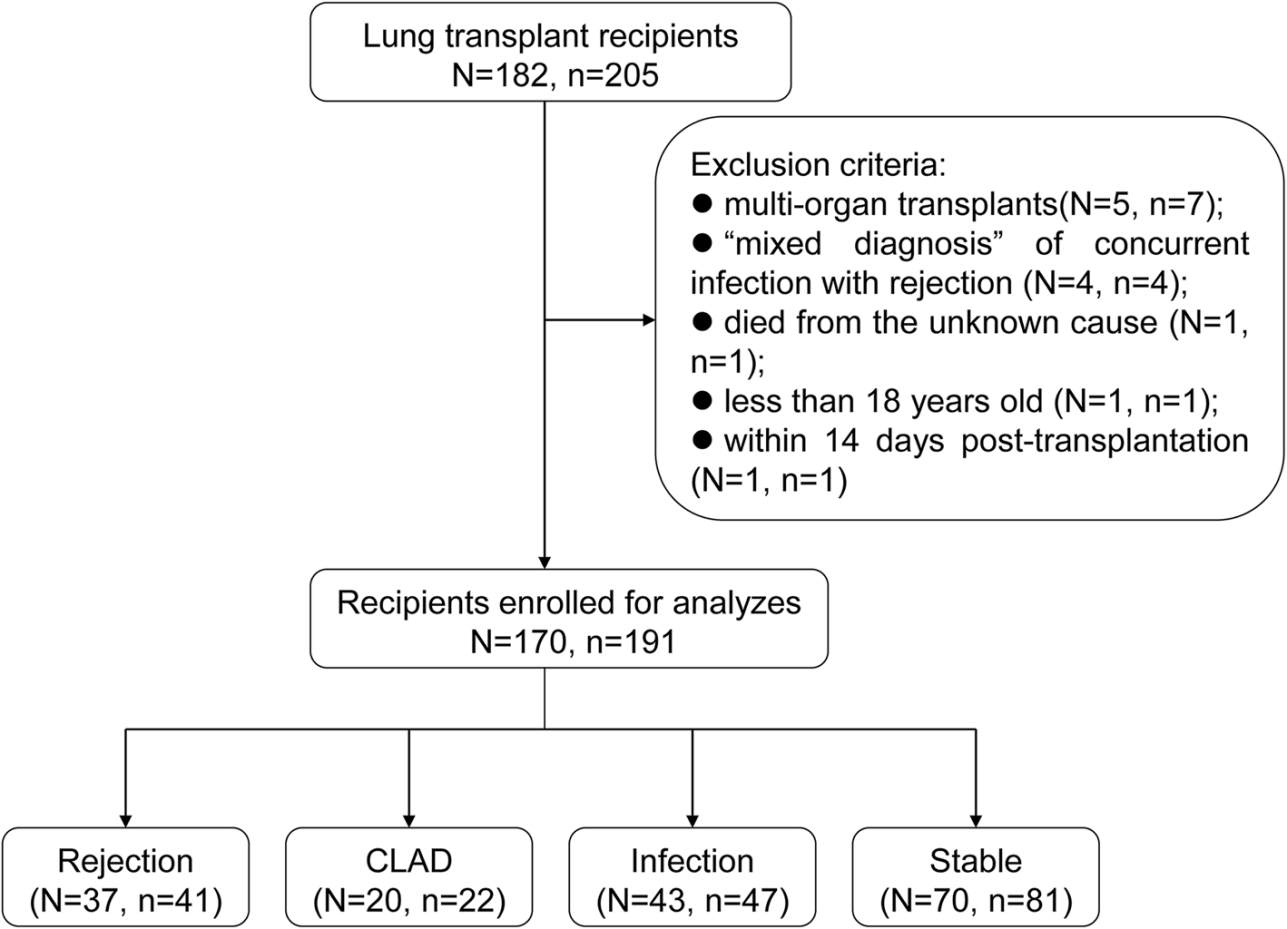
The flow chart of the study participants of this study. N = number of patients; n = number of samples

Excluding 14 blood samples from 12 recipients whose information was unsuitable for this study, 191 blood samples from 170 LTRs were included in this study. Among them, 151 blood samples were collected from 151 recipients, 36 from 18 recipients (the recipients had their cfDNA analyzed twice), and four from one recipient (the recipient had his cfDNA analyzed four times). Figure 1 shows a flow chart of the study participants. Demographic characteristic of the study participants was shown in Table 1. The primary indications for lung transplantation in this study included chronic obstructive pulmonary disease, interstitial lung disease, bronchiectasis, etc. Allograft function was maintained in each lung transplant recipient using a routine regimen of three immunosuppressants, including tacrolimus, a cell cycle inhibitor, and glucocorticoids.

**Table 1:** Basic characteristic and clinical description of lung transplant recipients

Demographics	Acute rejection (*n* = 37)	Infection (*n* = 43)	CLAD (*n* = 20)	Stable (*n* = 70)	*P*-value
Age, years	63.50 (49.50 to 67.75)	57.0 (49.0 to 66.0)	63.0 (57.25 to 69.50)	61.0 (55.0 to 66.0)	0.322
Gender					0.254
Male, n(%)	30 (81.08%)	30 (69.77%)	18 (90%)	57 (81.43%)	
Female, n(%)	7 (18.92%)	13 (30.23%)	2 (10%)	13 (18.57%)	
Body mass index (kg/m2)	19.01 ± 4.01	20.93 ± 3.57	21.25 ± 3.46	20.53 ± 3.62	0.069
Time from LTR to sample collection (Days)	39.0 (24.0 to 196.0)	404.0 (112.0 to 830.0)	920.50 (626.75 to 1856.50)	382.50 (74.75 to 799.25)	< 0.001
Type of transplantation					0.094
Single lung, n(%)	16 (43.24%)	22 (51.16%)	11 (55%)	47 (67.14%)	
Bilateral lung, n(%)	21 (56.76%)	21 (48.84%)	9 (45%)	23 (32.86%)	
PRA status					0.022
Positive ( +)	6 (16.22%)	2 (4.65%)	3 (15%)	2 (2.86%)	
Negative (-)	31 (83.78%)	41 (95.35%)	17 (85%)	68 (9.71%)	
FEV1%pred change rate (%)	-25.39 (-39.56 to -17.99)	-10.12 (-19.26 to -5.12)	-21.88 (-32.05 to -11.68)	-3.56 (-8.10 to -0.65)	< 0.001
FEV1/FVC% change rate (%)	-11.37 (-19.14 to -0.48)	-5.37 (-12.79 to 0)	-10.53 (-23.28 to 0.09)	-1.41 (-6.46 to 1.83)	< 0.001
FVC%pred change rate (%)	-22.0 (-40.7 to -13.07)	-7.69 (-18.13 to -0.16)	-19.71 (-29.69 to -10.17)	-3.25 (-9.80 to 0)	< 0.001
DLCO%pred change rate (%)	-16.57 (-37.93 to -10.22)	-7.76 (-15.20 to -1.05)	-19.73 (-23.83 to -6.36)	-2.0 (-8.35 to -1.90)	< 0.001
Prior episode of acute rejection, n (%)	10 (27.01%)	3 (6.98%)	12 (60%)	1 (1.43%)	< 0.001
Gastroesophageal reflux disease, n (%)	0	0	3 (15%)	1 (1.43%)	0.001
Cytomegalovirus IgG status, n (%)					0.460
D + /R +	31(83.78%)	32(74.42%)	17(85%)	50(71.43%)	
D − /R −	2(5.41%)	5(11.63%)	1(5%)	7(10%)	
D + /R −	3(8.11%)	2(4.65%)	2(10%)	3(4.29%)	
D − /R +	1(2.7%)	4(9.3%)	0	10(14.29%)	
Indication for lung transplantation, n (%)					0.080
Bronchiectasis	4(10.81%)	1(2.33%)	1(5%)	4(5.71%)	
Chronic obstructive pulmonary disease	11(29.73%)	11(25.58%)	11(55%)	21(30%)	
Connective tissue disease related interstitial lung disease	2(5.41%)	6(13.95%)	0	3(4.29%)	
Idiopathic interstitial pulmonary disease	13(35.14%)	14(32.56%)	5(25%)	32(45.71%)	
Pulmonary lymphangioleiomyomatosis	2(5.41%)	3(6.98%)	0	0	
Pulmonary arterial hypertension	0	1(2.33%)	1(5%)	0	
Work-related lung diseases	1(2.7%)	1(2.33%)	0	6(8.57%)	
Others	4(10.81%)	6(13.95%)	2(10%)	4(5.71%)	

### The levels of plasma dd-cfDNA

The demographics of the study recipients who were stratified and divided into groups at the time of diagnosis were shown in Table 1. There was no significant difference in age and gender ratio among the four group (*p* = 0.322 and 0.254, respectively).

**Fig. 2:**
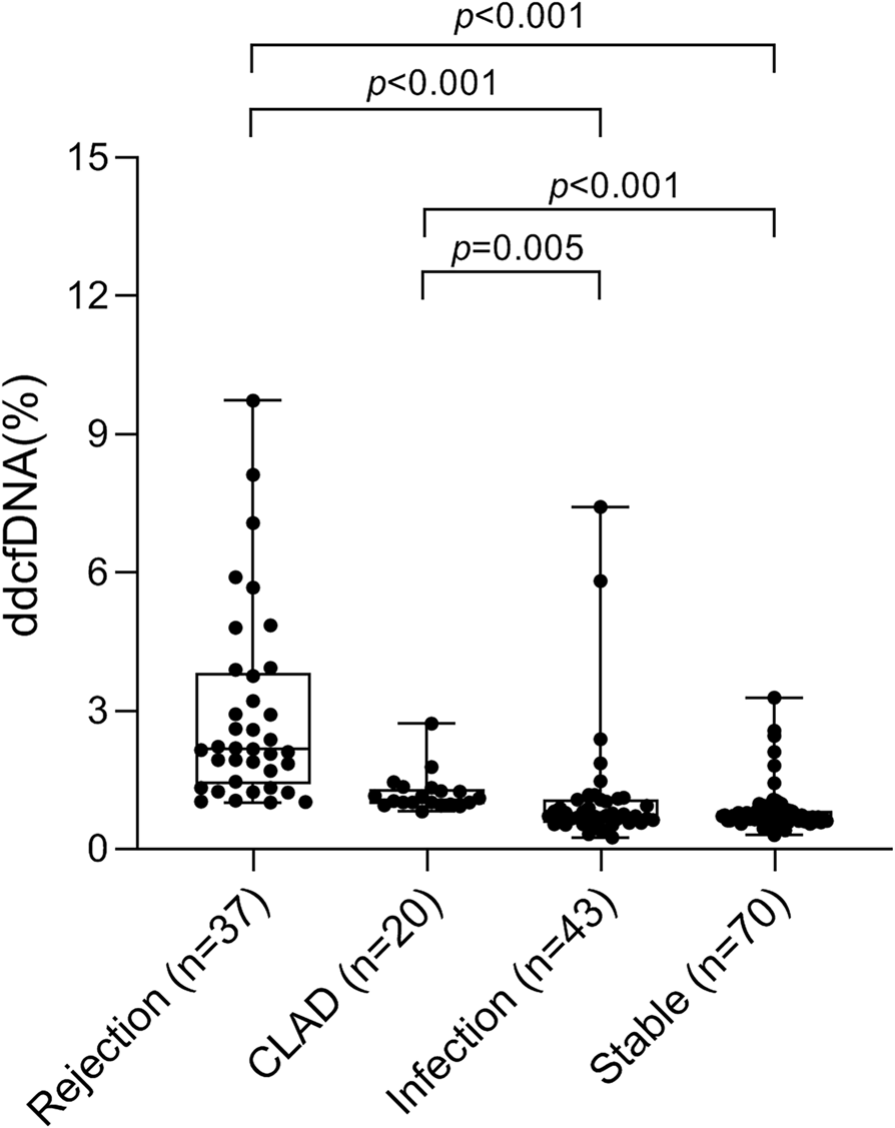
Box plots representing median and 25-75th quartiles (interquartile range) for dd-cfDNA levels associated with acute rejection, infection, CLAD and stable healthy

Kruskal–Wallis test showed that there were significant differences in the level of dd-cfDNA (%) among the 4 groups, with *p* < 0.001 (Fig. 2). Among them, the level of dd-cfDNA (%) was highest (median 2.17, IQR [1.40–3.82]) in AR group, higher in CLAD group (median 1.07, IQR [0.98–1.31]), lower in infection group (median 0.71, IQR [0.57–1.07]), and lowest in stable group (median 0.71, IQR [0.61–0.84]). Log rank test showed that there was no significant difference in dd-cfDNA levels between stable group and infection group, with *p* > 0.999. Also, no significant difference was found in dd-cfDNA levels between AR group and CLAD group, with *p* = 0.118. However, dd-cfDNA levels were significantly higher in AR group when compared to infection group and stable group, with *p* < 0.001 and *p* < 0.001, respectively. In addition, plasma dd-cfDNA levels were significantly higher in CLAD group when compared to infection group and stable group, with *p* = 0.005 and *p* < 0.001, respectively.

The Kruskal–Wallis test showed that there was no significant difference between LTRs with single- or bilateral lung transplantation in the stable group (Z = -0.013, *p* = 0.990), with the level of dd-cfDNA (%) being (median 0.71, IQR [0.60–0.86]) in LTRs with single lung transplantation and (median 0.69, IQR [0.61–0.98]) in those with bilateral lung transplantation.

### The performance of plasma dd-cfDNA levels

**Fig. 3:**
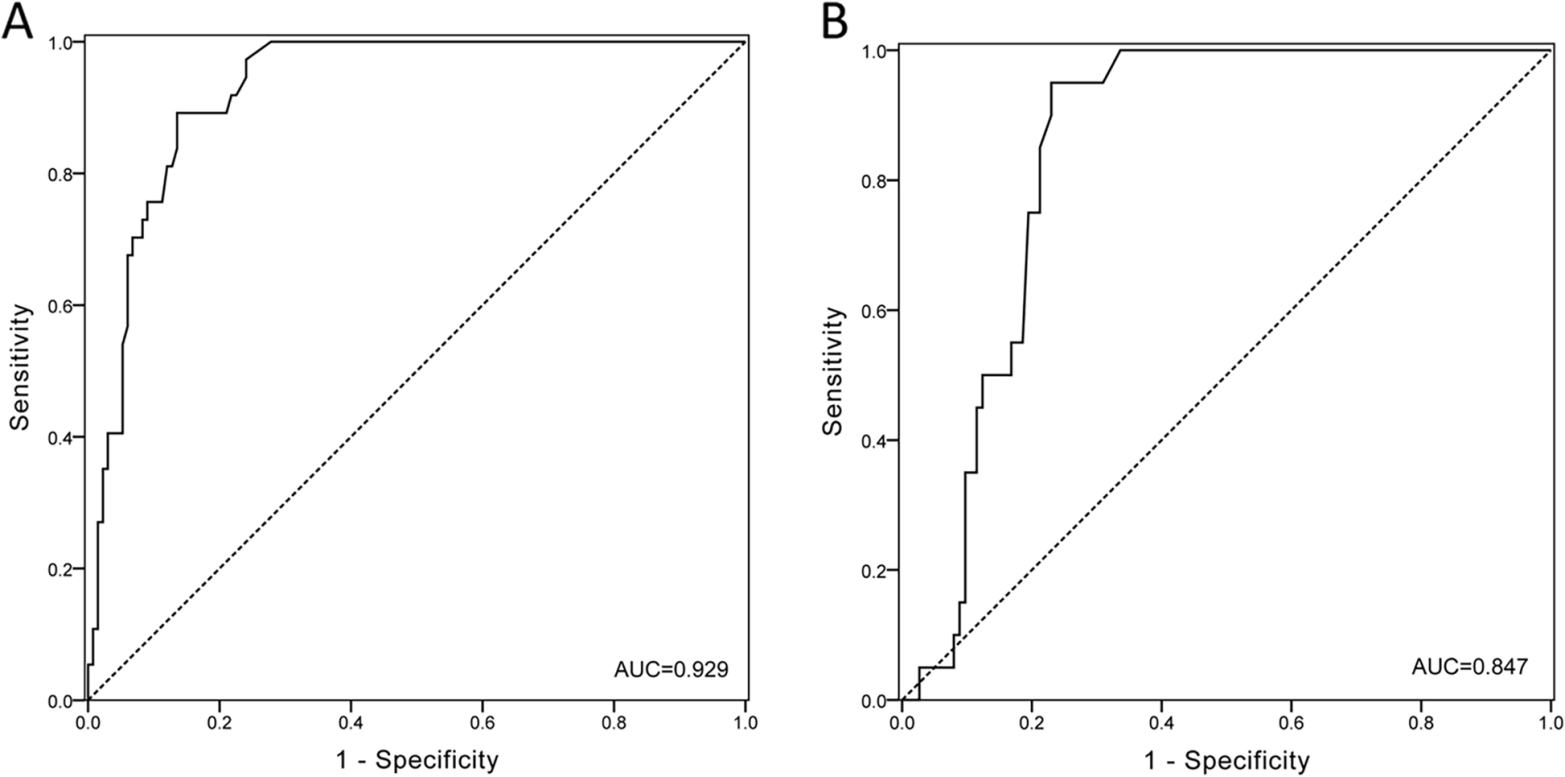
ROC curve for the performance of plasma dd-cfDNA. **A** ROC analysis of the performance of dd-cfDNA in classifying AR and the other 3 groups (including CLAD, infection and stable normal groups). **B** ROC analysis of the performance of dd-cfDNA in determine CLAD and infections as well as stable

The area under the curve (AUC) of the receiver operator characteristics (ROC) analysis was applied to evaluate the performance of the dd-cfDNA (%) levels in diagnosing AR and CLAD, respectively. The ROC curve demonstrated 0.929 (95%CI, 0.892–0.967; *p* < 0.001), where the optimal threshold of 1.17% was determined for AR (Fig. 3A). At this threshold, the sensitivity for AR diagnosis was 89.19% (95% CI, 74.6–97.0) and specificity was 86.47% (95%CI, 79.5–90.6), with positive predictive level (PPV) being 64.7% (95% CI, 50.4–74.1), and negative predictive level (NPV) being 96.6% (95% CI, 91.9–98.6), respectively.

Figure 3B showed that the AUC-ROC of the dd-cfDNA level (%) in discriminating of CLAD from infection and stable groups demonstrated an AUC of 0.847 (95% CI, 0.783–0.911; *p* < 0.001), where the optimal threshold of 0.89% was determined for dd-cfDNA. At this cutoff, sensitivity for CLAD was 95% (95%CI, 75.1–99.9) and specificity was 76.99% (95% CI, 68.1–84.4), with PPV of 42.2% (95%CI, 33.9–51.0) and NPV of 98.8% (95%CI, 92.8–99.8).

### The dynamic change of the dd-cfDNA levels

**Fig. 4:**
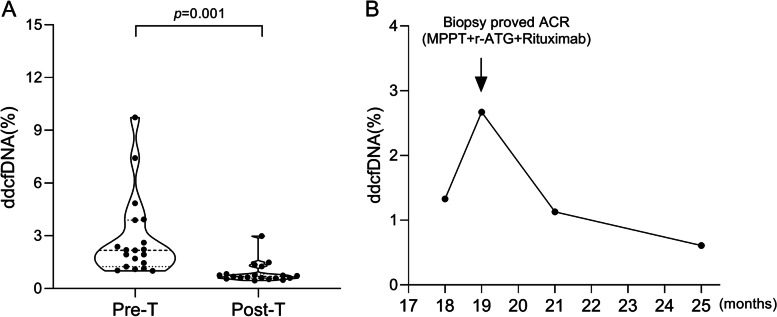
Dynamic monitoring of dd-cfDNA assisted LTRs treatment for decreased lung function. **A** Dynamics of dd-cfDNA before and after the treatment for LTRs, the black dot represents the results of two dd-cfDNA tests pre-treatment (Pre-T) and post-treatment (Post-T). **B** dd-cfDNA (%) and pathological monitoring of an ACR patient during treatment, the black dots represent the results of four dd-cfDNA tests during the treatment, and the arrows indicate the time point when AR was diagnosed and anti-rejection treatment was performed

In addition, 19 recipients had their plasma dd-cfDNA (%) detected repeatedly, with 18 recipients detected twice at different period of post-transplant time and one recipient detected longitudinally 4 times at specified intervals post-transplant. Among the 18 recipients, 14 were diagnosed with AR, 2 with CLAD, and 3 with infection. Paired t test demonstrated that there was significant difference in dd-cfDNA (%) before and after the treatment (2.84 ± 2.31 vs. 0.87 ± 0.59, *p* = 0.001) among the patients. The levels of dd-cfDNA were significantly decreased after the treatment, with the levels down below the cut-off level of 1.17% in ten of the patients who underwent AR treatment. At the same time, the clinical symptom for AR was relapsed (Fig. 4A).

For one recipient whose plasma dd-cfDNA was detected longitudinally four times, the thoracic CT was scanned repeatedly (Figs. S1, S2 and S3) at the same time with plasma dd-cfDNA detection. The level of dd-cfDNA detected was 1.33% for the first time though the recipient hadn’t shown any clinical symptom or manifestations at that time when it was one-and-a-half-year post-lung transplantation. However, 1 month later, the patient experienced an acute injury exacerbation, who suffered cough, chest tightness and progressive shortness of breath. Thoracic CT images later showed multiple infiltration and shadows in the right allograft lung. The patient had a sharp drop in lung function at the initial stage of the exacerbation episode, with progressing respiratory failure within several days. The comprehensive information didn’t show any infectious indication for his exacerbation in the clinical settings. The level of dd-cfDNA was elevated to 2.67% at that time. Thus, the patient was diagnosed as proven ACR by histopathology through TBLB. Based on the diagnosis, we changed the treatment strategy and prescribed reinforced immunosuppressive medications for the patient, with pulsed methylprednisolone, rabbit anti-human T lymphocyte immunoglobulin (r-ATG), and rituximab in combination. The symptom disappeared gradually after he received the reinforced immunosuppressive treatment, and the level of dd-cfDNA was lowered down to 1.13% after 2 months and further to 0.78% after 4 months, respectively. Figure 4B showed the dynamic change of the dd-cfDNA of the patient from the initial to the end of the exacerbation episode.

### Correlation of dd-cfDNA levels with the change rate of FEV1%pred

**Fig. 5:**
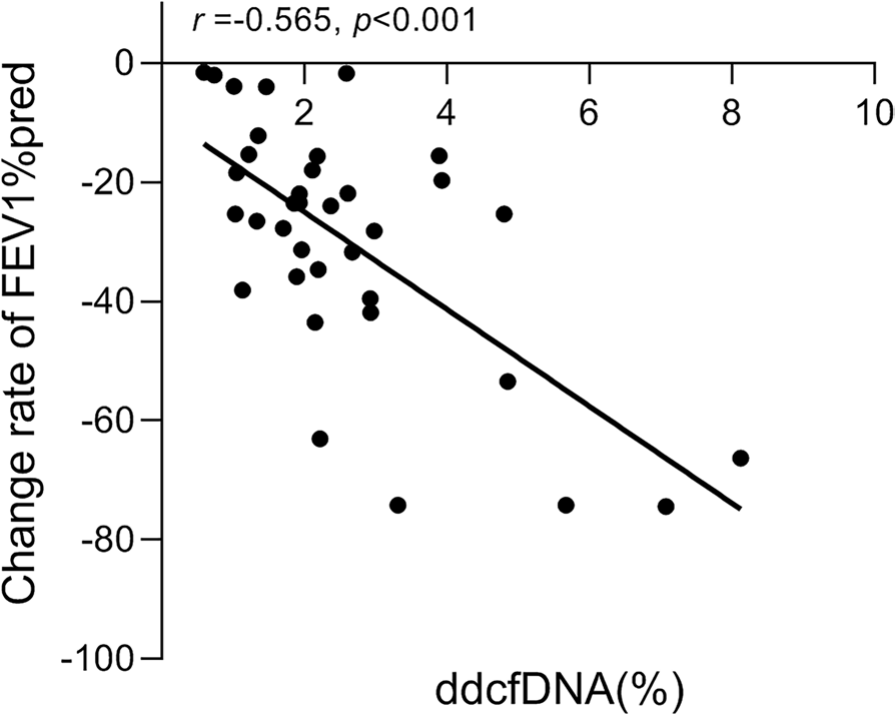
Correlation of dd-cfDNA levels with the change rate of FEV1%pred in AR group

In addition, Paired *t*-test showed that lung function was significantly decreased in all group recipients except stable group recipients when compared to their previous baseline lung function (*p* < 0.001). Post-hoc analysis showed that there were significant differences in the change rate of FEV1%pred among the four groups (Table 1), significantly declined rate of FEV1%pred was found in AR, CLAD and infection group, when compared with that in stable group (*p* < 0.001, *p* = 0.013, and *p* < 0.001, respectively). Spearman correlation analysis showed that the change rate of FEV1%pred was significantly correlated with the level of dd-cfDNA (%) in AR group (*r* = -0.565, *p* < 0.001, Fig. 5).

## Discussion

In this study, plasma samples were used to evaluate the performance of dd-cfDNA levels in diagnosing allograft lung rejection. The detection of dd-cfDNA levels has been validated in other solid organ transplantations internationally in recent years, while this is the first study to explore its application in lung transplantation among Chinese patients. Our results showed that the level of dd-cfDNA was significantly elevated in both AR and CLAD groups than stable group after unilateral and bilateral lung transplantation. Moreover, we observed that the dynamic change of dd-cfDNA level was consistent with the clinical sign of rejection as well as treatment response for rejection. Although the application of plasma dd-cfDNA value has been applied in the diagnosis of lung allograft rejections in few new published studies, this study is the first to combine dd-cfDNA level with clinically comprehensive information such as clinical signs, lung function test results, CT images and treatment response.

The results from this study showed that there were significant differences in the plasma dd-cfDNA (%) levels among the 4 groups, with the highest level in AR group. The ROC curve demonstrated an AUC of 0.929, where the optimal threshold of 1.17% was determined for AR, with the sensitivity for AR diagnosis been 89.19% (PPV = 64.7%, NPV = 96.6%). The study design and result of this investigation were very similar to that by Kiran et al. [14], but in their study, the plasma dd-cfDNA (%) level was highest in CLAD group, and higher in AR group. The likely reason for the very small difference in plasma dd-cfDNA (%) levels might be the racial differences in the participants between the two studies. Our suggestion is supported by a recent study in which Williams et al. [29] showed that rejection among African American recipients was associated with lower dd-cfDNA values compared to non-African American recipients who had received renal transplantation.

The other alternative explanation could be attributed to the limited sample size in both studies. Nevertheless, in this study, we suggest employing plasma dd-cfDNA (%) as a non-invasive biomarker for the clinical diagnosis of AR. This suggestion can be supported by growing number of recent studies in lung transplantation [30, 31] and other solid organ transplantation [12–16, 32, 33].

Moreover, Wilcoxon rank sum tests for cohorts with CLAD showed a significantly elevated dd-cfDNA (%) level compared with the stable cohort. AUC-ROC of the dd-cfDNA value (%) was applied to discriminate CLAD from CLAD-free cohort, which demonstrated an AUC of 0.874, where the optimal threshold of 0.89%, with the sensitivity of 95% and specificity of 76.99% (PPV = 42.2%, NPV = 98.8%). The result presented in this study was consistent with those of recent studies [19, 30–32, 34] and are supported by the recent research on the other solid organ transplantation [12–16, 32, 33]. Compared to other solid organ transplantation, the long-term outcomes of lung transplant remain poor, which is largely due to CLAD [25, 26]. Since there is no effective treatment to reverse CLAD, it is critically important to prevent it through early detection, and to create targeted strategies to slower its progress. Therefore, establishing diagnostic or prognostic biomarkers for CLAD diagnosis or prediction is crucial to improve long-term survival. Although AR is usually diagnosed by histopathology with serial TBLB [35, 36], this diagnostic method has limited reliability, and is invasive and risky [37, 38]. In contrast to the disadvantages of the commonly used invasive procedures in diagnosing lung allograft injury (including AR + CLAD) [39, 40], the results of this study suggests that dd-cfDNA can be employed as a safe non-invasive biomarker in determining a spectrum of lung rejections including acute and chronic rejection.

Moreover, we observed that the elevated dd-cfDNA level at initial stage of the AR episode was lowered gradually with the treatment against AR in one patient who had been diagnosed of AR. By detecting of dd-cfDNA levels repeatedly, before and after the clinically-based treatment, the study found that the elevated dd-cfDNA levels were significantly lowered down following the treatment (Fig. 4A). In addition, by monitoring the dynamic change of the dd-cfDNA level, we found that the dd-cfDNA level was consistent with but much more sensitive than the clinical signs including lung function change and CT image manifestations in diagnosing AR and CLAD. Take an example of a recipient from AR group in our study, whose plasma dd-cfDNA was assayed longitudinally four times, the thoracic CT was scanned repeatedly at the same time with plasma dd-cfDNA detection. The level of dd-cfDNA detected was 1.33% for the first time, even though the recipient hadn’t shown any clinical symptom or manifestation at that time. However, one month later, the patient suffered severe cough, and then, he suffered chest tightness and progressive shortness of breath, with thoracic CT images showed multiple infiltrations and lesions, ground glass opacities, and interlobular septal thickening in the right allograft lung. The level of dd-cfDNA was elevated to 2.67% at that time. Until that time, the patient was diagnosed as AR and received the anti-rejection therapy with reinforced immunosuppressants. Both the symptom and CT images was improved gradually after he received the reinforced immunosuppressive treatment, and the level of dd-cfDNA was lowered gradually with time. Our results suggested that plasma dd-cfDNA has an important role in helping physicians not only to monitor the allograft injury but also to give some indication for clinician to determine the appropriate treatment strategy. Similar study suggestions have recommended in the other solid organ transplantation.

The dd-cfDNA level for the cohort with respiratory allograft infection in the absence of concurrent rejection was significantly lowered when compared to the AR and CLAD cohort, and it was slightly elevated when compared with stable healthy cohort, although it did not achieve statistical significance. The study result was in agreement with that of Kiran et al. [19], which also failed to find a significant difference in the plasma dd-cfDNA level between infection and stable group. Since plasma dd-cfDNA represents the allograft tissue injury, it should be slightly elevated in these infection cohort recipients. A further study with larger samples should therefore be performed to explore the value of dd-cfDNA level in the application of allograft infection.

In order explore the association of plasma dd-cfDNA level with the other non-invasive index for allograft injury, we analyzed its correlation with lung function. The results showed that there was a significant negative correlation between plasma dd-cfDNA level and FEV1% pred change rate in AR group. The results were expected because allograft function should be decreased with the occurrence of injury (AR). Level of dd-cfDNA has no significant correlation with FEV1% change rate in CLAD group. Because multiple factors contribute to CLAD, including alloantigen-dependent (cellular and antibody-mediated rejection) and alloantigen-independent factors (infection, aspiration, ischemia, and autoimmunity). Increase level of dd-cfDNA main related to alloantigen-dependent allograft-injury. However, lung function is not a specific index, as pulmonary function decreases in many kinds of pulmonary complications among LTRs. Our study results suggest that plasma dd-cfDNA is an interesting biomarker of rejection (including acute and chronic rejections), which has a much higher specificity than lung function in diagnosing of allograft rejection.

Collectively, our study results, for the first time, showed interesting findings in Chinese lung transplantation by using a total of 6,200 human SNP loci to select and detect. Compared to other already available cf-ddDNA kits (Multiplex PCR based sequencing) such as Alloseq or AlloSure, our method can obtain more effective SNP loci suitable for Chinese population to ensure the accuracy of dd-cfDNA test. Multiplex PCR based sequencing methods cannot remove PCR duplicate, thus, many SNPs were employed in our study for the following reasons: 1) dd-cfDNA quantification is affected because of PCR bias; 2) The effective depth of sequencing is not known, and the limit of detection (LOD) cannot be accurately evaluated; 3) The ability to remove the wrong base of sequencing or PCR error is limited; 4) the limit of blank (LOB) is high.

It should be noted that the present study has a few limitations. First, it was a single center study with a limited sample size, and a larger multicenter study is needed in China to accurately evaluate the diagnostic value of plasma dd-cfDNA in diagnosing rejection in lung transplantation. Second, this was a cross-sectional study in which samples were randomly collected from some patients randomly. Due to the high cost of dd-cfDNA assay, no longitudinal comparison was performed on schedule. However, dd-cfDNA detection should be conducted before and after the treatment to confirm the diagnostic value of dd-cfDNA during active allograft rejection in lung transplant recipients. A prospectively designed study should be performed in order to help clinician develop intervention strategies for CLAD at the early stage. Third, BALF should be a good sample to study, as the level of dd-cfDNA in BALF might provide researchers with more information when combined with the level in plasma. A further study on dd-cfDNA in BALF is now ongoing which will be reported in the near future. Finally, lung biopsy was not available from every participant in this study, thus there might be some bias in the clinical diagnosis. However, treatment response helped a lot when the study initiated and the clinical information was checked and reviewed.

## Conclusion

In conclusion, plasma dd-cfDNA is significantly elevated in patients with AR and CLAD following lung transplantation, and it is more prominent during the period of AR. Plasma dd-cfDNA assessment therefore holds promise as a noninvasive biomarker for early detection of lung allograft injuries including AR and CLAD, with a high sensitivity and specificity.

## Supplementary Information

**Additional file 1: Figure S1.** Thoracic computed tomography (CT) images from a right single lung transplant recipient during different period post lung transplantation.

**Additional file 2: Figure S2.** Thoracic CT images show multiple infiltration in the right allograft lung during the period of the acute exacerbation.

**Additional file 3: Figure S3.** Thoracic CT images during the recovery stage of the acute exacerbation.

**Additional file 4: Table S1.** Information on the infectious agents isolated from BALF in the AR group.

**Additional file 5: Table S2.** Information on the infectious agents isolated from BALF in the infection group.

## Data Availability

The datasets generated and/or analysed during the current study are available in the [NCBI] repository, [https://dataview.ncbi.nlm.nih.gov/object/PRJNA801094?reviewer=vap7nq272jc2l86ligrr7clm3m (accession no. PRJNA801094)].

## Abbreviations

ACR: Acute cellular rejection
AMR: Antibody mediated rejection
AR: Acute rejection
Bas: Basiliximab
BALF: Bronchoalveolar lavage fluid
CMV: Cytomegalovirus
CT: Computed tomography
CTD: Connective tissue disease
dd-cfDNA: Donor-derived cell-free DNA
DSA: Donor specific antibody
LTRs: Lung transplant recipients
M: Months
N: Number of patients
n: Number of samples
PRA: Panel reactive antibody
TBLBs: Trans-bronchial lung biopsies
