# Supplementary material for: Application of plasma donor-derived cell free DNA for lung allograft rejection diagnosis in lung transplant recipients
Source: BMC Pulm Med. 2023 Jan 26;23:37. doi: 10.1186/s12890-022-02229-y (PMC9881379; doi:10.1186/s12890-022-02229-y)
Supplement: Supplementary file 1 — Additional file 1: Figure S1. Thoracic computed tomography (CT) images from a right single lung transplant recipient during different period post lung transplantation. [file 12890_2022_2229_MOESM1_ESM.docx]

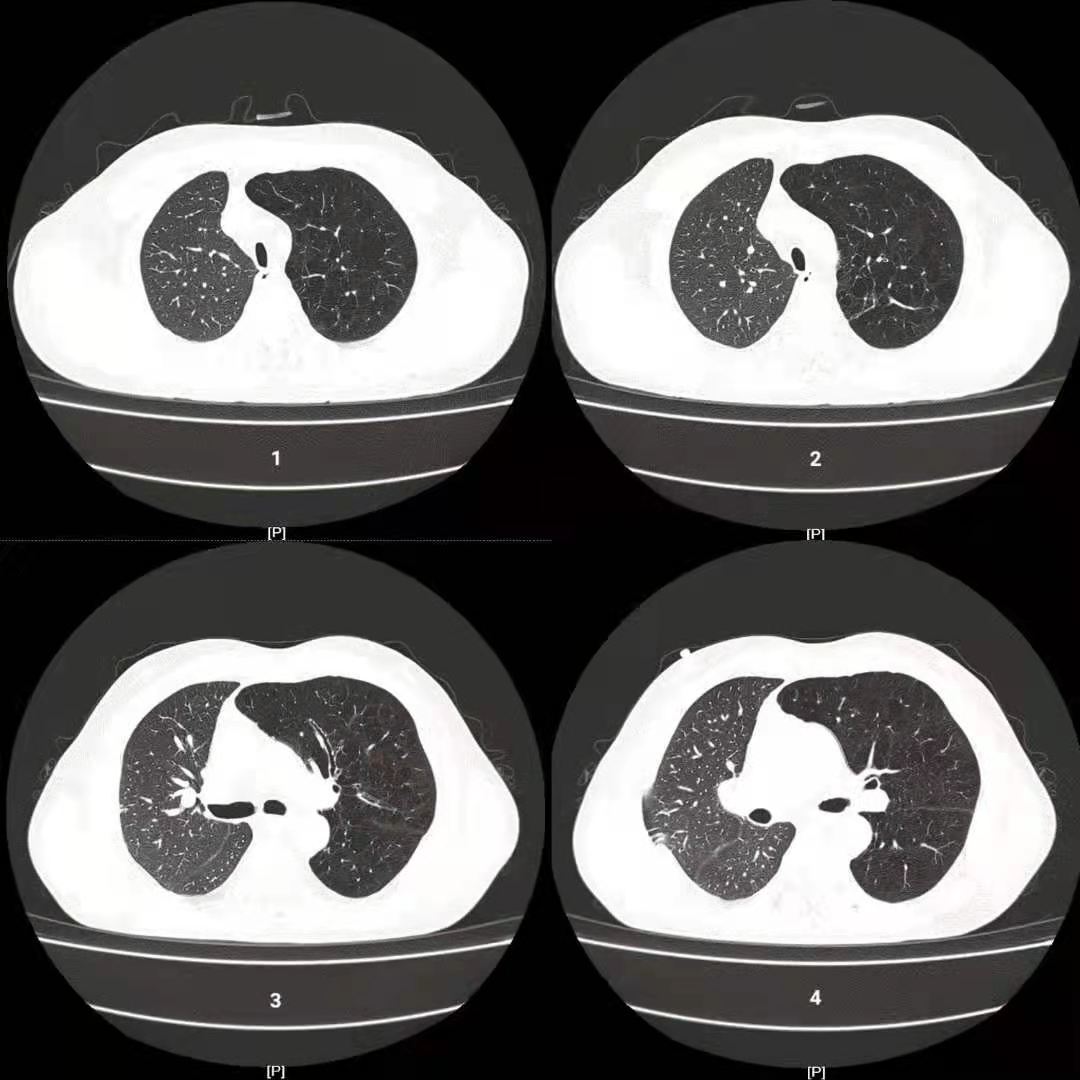


**Figure S1.** Thoracic computed tomography (CT) images from a right single lung transplant recipient during different period post lung transplantation. A: Thoracic CT images shows there was no any infiltration in the allograft right lung or the native left lung. The recipient was in clinically stable condition when was one-and-a-half-year post-lung transplantation, and the level of cf-DNA was 1.33% at that time.
