# Supplementary material for: Application of plasma donor-derived cell free DNA for lung allograft rejection diagnosis in lung transplant recipients
Source: BMC Pulm Med. 2023 Jan 26;23:37. doi: 10.1186/s12890-022-02229-y (PMC9881379; doi:10.1186/s12890-022-02229-y)
Supplement: Supplementary file 2 — Additional file 2: Figure S2. Thoracic CT images show multiple infiltration in the right allograft lung during the period of the acute exacerbation. [file 12890_2022_2229_MOESM2_ESM.docx]

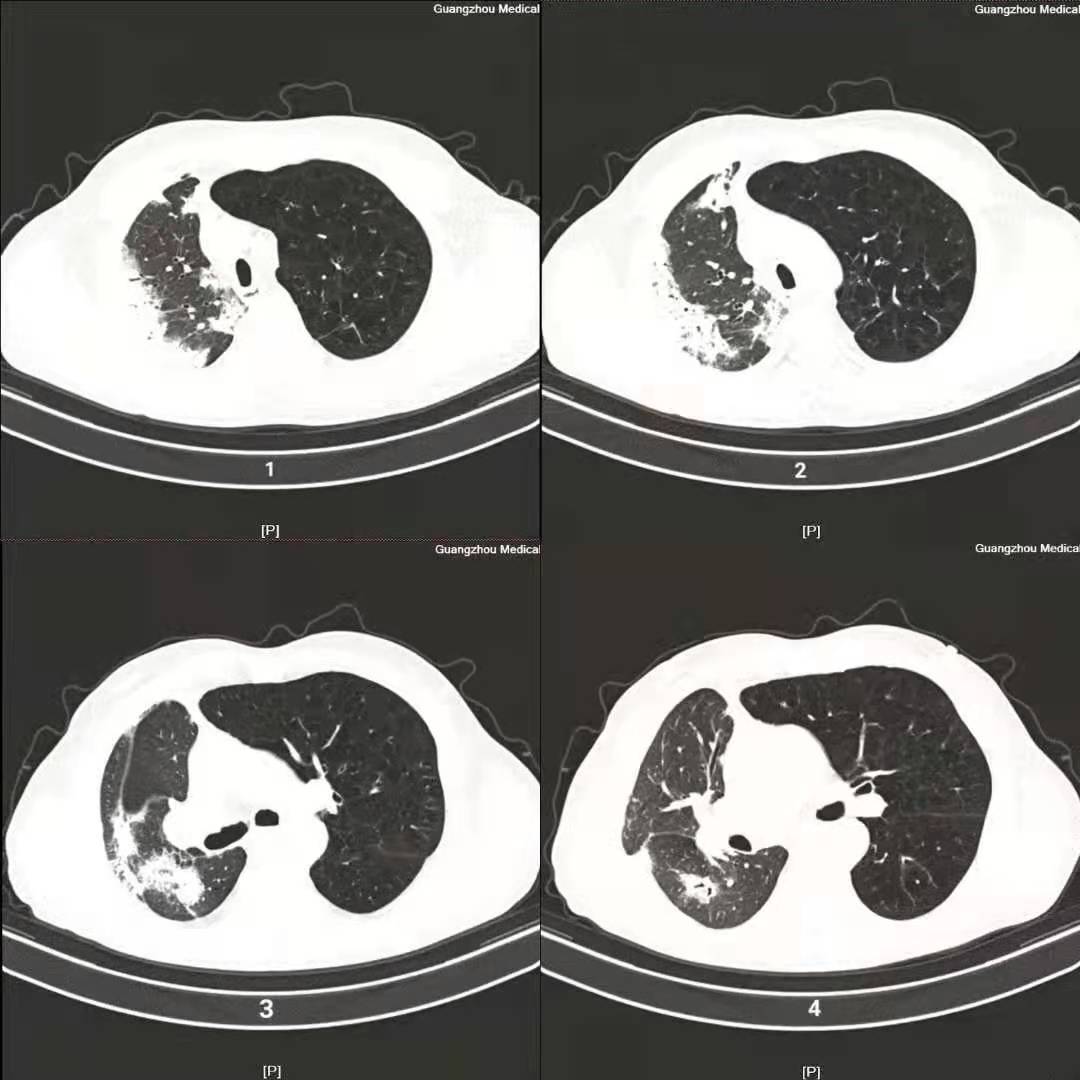


**Figure S2.** Thoracic CT images show multiple infiltration in the right allograft lung during the period of the acute exacerbation, when was one-year and seven months post-lung transplantation, and the value of cf-DNA was 2.67% at that time. The patient was diagnosed as proven acute rejection by histopathology, and the lung tissue was gotten by TBLB guided by CT images from the infiltration position of the right allograft lung.
