# Supplementary material for: Application of plasma donor-derived cell free DNA for lung allograft rejection diagnosis in lung transplant recipients
Source: BMC Pulm Med. 2023 Jan 26;23:37. doi: 10.1186/s12890-022-02229-y (PMC9881379; doi:10.1186/s12890-022-02229-y)
Supplement: Supplementary file 3 — Additional file 3: Figure S3. Thoracic CT images during the recovery stage of the acute exacerbation. [file 12890_2022_2229_MOESM3_ESM.docx]

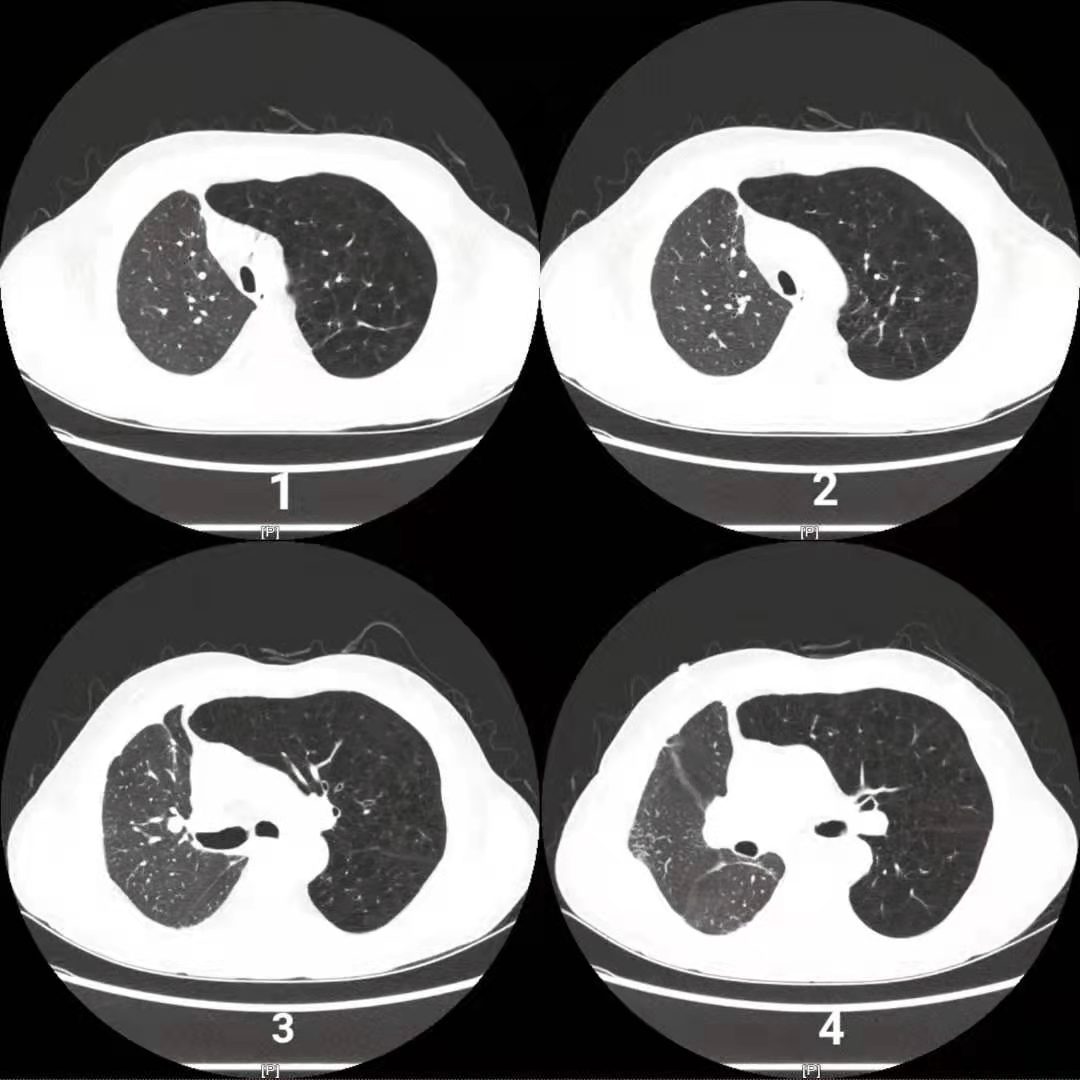


**Figure S3.** Thoracic CT images during the recovery stage of the acute exacerbation, when was 4 months later from the episode of the acute exacerbation, after the patient received the reinforced immunosuppressive treatment. The value of cf-DNA was 1.13% at that time. The symptom had disappeared gradually during that period.
