# Supplementary material for: Application of plasma donor-derived cell free DNA for lung allograft rejection diagnosis in lung transplant recipients
Source: BMC Pulm Med. 2023 Jan 26;23:37. doi: 10.1186/s12890-022-02229-y (PMC9881379; doi:10.1186/s12890-022-02229-y)
Supplement: Supplementary file 4 — Additional file 4: Table S1. Information on the infectious agents isolated from BALF in the AR group. [file 12890_2022_2229_MOESM4_ESM.docx]

**Table S1.** The information about infectious agents isolated from BALF among AR group.

| Number | cf-DNA  (%) | Group  (n=41) | Isolated pathogen |
| --- | --- | --- | --- |
| 29 | 3.75% | AR | negative |
| 196 | 2.37% | AR | negative |
| 3 | 1.33% | AR | negative |
| 123 | 2.17% | AR | negative |
| 39 | 3.21% | AR | negative |
| 106 | 2.93% | AR | negative |
| 134 | 2.11% | AR | negative |
| 46 | 1.24% | AR | negative |
| 108 | 2.92% | AR | negative |
| 159 | 1.46% | AR | Stenotrophomonas colonization |
| 193 | 1.89% | AR | negative |
| 86 | 3.89% | AR | negative |
| 153 | 2.19% | AR | negative |
| 98 | 2.15% | AR | Acinetobacter baumannii colonization |
| 163 | 1.93% | AR | negative |
| 204 | 2.59% | AR | negative |
| 216 | 1.01% | AR | negative |
| 202 | 1.05% | AR | negative |
| 45 | 1.02% | AR | Pseudomonas aeruginosa colonization |
| 139 | 4.80% | AR | negative |
| 160 | 2.06% | AR | negative |
| 103 | 1.23% | AR | Pseudomonas aeruginosa colonization |
| 177 | 1.85% | AR | negative |
| 226 | 1.22% | AR | negative |
| 113 | 2.98% | AR | negative |
| 205 | 5.90% | AR | negative |
| 211 | 6.26% | AR | negative |
| 219 | 1.96% | AR | negative |
| 225 | 3.31% | AR | negative |
| 184 | 0.85% | AR | negative |
| 73 | 1.03% | AR | negative |
| 36 | 1.70% | AR | negative |
| 8 | 8.12% | AR | negative |
| 21 | 1.93% | AR | negative |
| 130 | 5.67% | AR | Candida albicans colonization |
| 17 | 1.33% | AR | negative |
| 24 | 2.67% | AR | negative |
| 138 | 1.35% | AR | negative |
| 32 | 1.13% | AR | negative |
| 146 | 7.07% | AR | negative |
