# Supplementary material for: Application of plasma donor-derived cell free DNA for lung allograft rejection diagnosis in lung transplant recipients
Source: BMC Pulm Med. 2023 Jan 26;23:37. doi: 10.1186/s12890-022-02229-y (PMC9881379; doi:10.1186/s12890-022-02229-y)
Supplement: Supplementary file 5 — Additional file 5: Table S2. Information on the infectious agents isolated from BALF in the infection group. [file 12890_2022_2229_MOESM5_ESM.docx]

**Table S2.** The information about infectious agents isolated from BALF among infection group.

| Number | dd-cfDNA (%) | Group (n=47) | Main pathogens |
| --- | --- | --- | --- |
| 121 | 1.17% | INF | Acinetobacter baumannii and klebsiella pneumoniae |
| 49 | 0.72% | INF | Pseudomonas aeruginosa |
| 75 | 1.09% | INF | Cytomegalovirus |
| 11 | 0.59% | INF | Klebsiella pneumoniae and pseudomonas aeruginosa |
| 186 | 0.64% | INF | MDR-pseudomonas aeruginosa and aspergillosis |
| 35 | 0.57% | INF | pseudomonas aeruginosa and aspergillosis |
| 78 | 0.42% | INF | pseudomonas aeruginosa and escherichia cescherichia coli |
| 81 | 0.68% | INF | HaemopHaemophilus influenzae |
| 99 | 5.81% | INF | Cytomegalovirus |
| 100 | 1.03% | INF | Cytomegalovirus |
| 223 | 0.74% | INF | Staphylococcus aureus and Haemophilus influenzae |
| 128 | 1.07% | INF | Adenovirus |
| 77 | 0.82% | INF | Influenza virus |
| 34 | 0.32% | INF | Pseudomonas aeruginosa and aspergillosis |
| 107 | 0.63% | INF | Nocardia |
| 180 | 0.57% | INF | False streptococcus pneumoniae |
| 90 | 0.66% | INF | Acinetobacter baumannii |
| 114 | 0.70% | INF | Nontuberculosis mycobacteria |
| 227 | 0.86% | INF | Streptococcus pneumoniae |
| 64 | 0.67% | INF | Pseudomonas aeruginosa |
| 120 | 0.78% | INF | Haemophilus influenzae |
| 63 | 0.58% | INF | Haemophilus influenzae |
| 95 | 0.52% | INF | Pneumocystis japonicum |
| 157 | 1.86% | INF | Cytomegalovirus |
| 174 | 0.67% | INF | Aspergillosis |
| 214 | 0.77% | INF | Streptococcus pneumoniae |
| 115 | 0.80% | INF | Respiratory syncytial virus |
| 84 | 0.66% | INF | Acinetobacter baumannii and cyanobacteria marneferi |
| 172 | 1.45% | INF | Aspergillosis |
| 127 | 0.94% | INF | Cytomegalovirus |
| 152 | 2.04% | INF | Pseudomonas aeruginosa and aspergillosis |
| 92 | 2.18% | INF | Cytomegalovirus |
| 55 | 0.54% | INF | Acinetobacter baumannii |
| 87 | 0.53% | INF | HaemopHaemophilus influenzae |
| 167 | 0.59% | INF | Acinetobacter baumannii and aspergillosis |
| 54 | 0.68% | INF | pseudomonas aeruginosa and aspergillosis |
| 51 | 1.47% | INF | Cytomegalovirus |
| 59 | 2.38% | INF | Cytomegalovirus and pseudomonas aeruginosa |
| 27 | 0.89% | INF | Burkholderia cepacia and pseudomonas aeruginosa |
| 7 | 0.75% | INF | pseudomonas aeruginosa |
| 18 | 0.76% | INF | Tuberculosis |
| 44 | 0.45% | INF | Escherichia coli |
| 26 | 7.42% | INF | Cytomegalovirus |
| 6 | 0.62% | INF | Pneumocystis japonicum |
| 20 | 0.77% | INF | Pseudomonas aeruginosa |
| 15 | 0.25% | INF | Pneumocystis japonicum |
| 16 | 0.54% | INF | Pseudomonas aeruginosa |
| 14 | 0.40% | INF | Mycobacteria |

dd-cfDNA: donor derived cell-free DNA; INF=infection
